# Water in PHI Nanopores: Modeling Adsorption, Solvent Structure, and Thermodynamics

**DOI:** 10.1021/acsomega.3c03308

**Published:** 2023-07-13

**Authors:** Julian Heske, Thomas D. Kühne, Markus Antonietti

**Affiliations:** † Department of Colloid Chemistry, Max Planck Institute of Colloids and Interfaces, MPI Research Campus Golm, D-14424 Potsdam-Golm, Germany; ‡ Dynamics of Condensed Matter and Center for Sustainable Systems Design, Chair of Theoretical Chemistry, University of Paderborn, Warburger Str. 100, D-33098 Paderborn, Germany

## Abstract

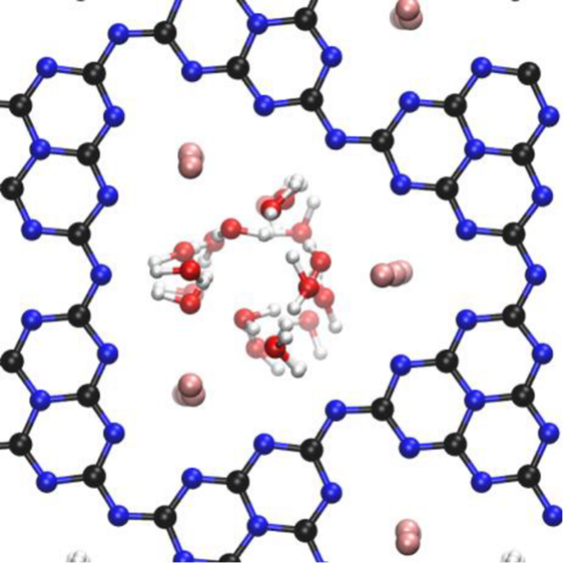

We modeled the uptake of water molecules into the nanopores of potassium-polyheptazineimide (K-PHI), a 2D covalent material that is one of the best water-splitting photocatalysts to date possessing experimentally reported strong water binding. In the current models, we find that first water molecules are bound with −94.5 kJ/mol, i.e., twice the cohesion energy of water and one of the highest adsorption enthalpies reported so far. This strong binding proceeds unexpectedly on a similar enthalpy level until the pore is filled, while the binding strength is passed through a conjugated water network. The tight binding is also expressed in calculated, strongly shortened O–O distances, which are on average about 5% shorter than in bulk water, which corresponds to a much higher water density, for a 2D structure above 1.1 g/ cm^3^. The H-bridges are strongly aligned in the direction perpendicular to the covalent planes, which could give reasons for the experimentally observed ultrahigh ion fluxes and conductivity of K-PHI membranes. Decomposition of the adsorption energy into components reveals an unexpectedly high charge transfer contribution, where the partly naked K^+^ ions play a key role. The latter fact not only offers a new structural lead motif for the design of more strongly, but reversibly binding adsorption materials involving metal ions on their surface but also puts cations as known cofactors in enzymes into a new light.

## Introduction

Water is from all possible views a special solvent. It is omnipresent on Earth, the fluid of life, and it is important for us beyond imagination. It is, however, presumably also the most mysterious solvents of all: water shows around 70 physico-chemical anomalies (depending on counting), its solid-state polymorphism is incredibly rich (ca. 26 different structures, depending on counting), and even the liquid state seems to be polymorphic. A mixture of two polymorphs is currently discussed to describe at least some of the anomalies.^1^

For instance, water has one of the smallest indices of refraction of all solvents (*n*_D_ = 1.33), which corresponds to a very low optical polarizability, just slightly above methane (*n*_D_ = 1.27). On the other hand, it has a very high dielectric constant (ε(20 °C) = 80.4), which however quickly “melts” with temperature and the related thermal weakening of H-bridges; i.e., it is the supramolecular water structure being responsible for the high dielectric constant and water polarity. Water also has a very high viscosity when compared to its apparently low molecular weight; i.e., liquid water is always dominated by water clusters. Changing water clusters and changing water structure will thereby allow to create new solvent properties and a special water.

Water structure can be changed by solutes and along material interfaces. H-bridge donors or acceptors realign the water structure, an effect which propagates into the liquid state for a distance of a few nanometers.^2,3^ So, can we change solubility and even pH or chemical reactivity of water as a solvent by addition of interacting compounds or here by confining its liquid state into pores with strongly H-bridge interacting pore walls? The answer is yes, as we know from the famous Hofmeister series.^4^ Hydrophobic compounds or proteins can be salted out of or salted in water, the melting points of DNA or proteins can change, enzymes show a different reactivity, and so on, all in the presence of salt. On the other hand, adding hydrophobic polymer electrolytes can result in water activity coefficients well above 1;^5^ i.e., water is chemically more reactive when it is not a part of bulk water.

Water filling a continuous pore system with one or two minor dimensions of the size of some water molecules is such a modified “nanowater”, here however free of disturbing solutes, and we expect that all known bulk properties and reactivities are changing. Limbach and co-workers in early work^6^ used ^1^H-spectroscopy of water in two different porous silicas (SBA15 and MCM41) to analyze the electron density at the water proton position and found rather strong shifts visible for up to rather high water fractions. Later work analyzed freezing, ice structure, and thermal expansion and also found relevant differences between bulk water and nanopore water.^7^

The intention of the current work is to move such work from silicas to porous systems with much stronger H-bridge donor and acceptor properties to extend the limits of the resulting water modification. Polyheptazineimides (PHIs) are stacked, regular covalent organic frameworks, with a comparably big pore in the structure.^8,9^ These triangular pores are lined by imide linkages (proton donors) and heptazinic nitrogen edges (proton acceptors). From these and similar systems, it is known that water cannot be removed even by extended vacuum drying,^10^ while liquid water and small cations rather flow freely through such pores.^11^

Modeling water in silico in such pores is thereby a helpful tool to understand physical and thermodynamic properties of such water. Binding enthalpy onto pore walls is one of the results, to be compared with the heat of evaporation of liquid water (40.65 kJ/mol). In addition, details of the bonding structure in between wall functionality and water and then water and water can be revealed. Van der Waals interactions are notoriously small in water (the nearby methane has 8.17 KJ/mol heat of evaporation), and the difference in evaporation is usually assigned to the number and the strength of the hydrogen bridges. Computer modeling allows us to quantify charge transfer highly, i.e., how much of an ion charge is distributed along the water network, thereby adding Coulombic components to the cohesion energy.

As PHIs are very powerful photo-, electro-, and chemocatalysts, we hope to deduce, as a result of such computer models, also relevant statements about the chemical reactivity of water in the pore system, such as potential acidicity or basicity, the electrochemical window of confined water, or co-solubilities of relevant gases to be photoreacted.

## Results and Discussion

The computational details are discussed first. We established a defect-free K-PHI model using experimental lattice parameters obtained by powder X-ray diffraction measurements^12^ and optimized the atomic positions with respect to energy. Then, we added single water molecules and analyzed structure, thermodynamics, and interaction contributions. Then, we extended the work to a higher number of water molecules to start to elaborate on the properties of “nanowater”.

Periodic density functional theory calculations were carried out using the hybrid Gaussian and plane wave approach,^13^ as implemented in the CP2K/Quickstep code.^14^ The Kohn–Sham orbitals were described by an accurate molecularly optimized double-zeta basis set with one additional set of polarization function, while the charge density was represented by plane waves with a density cutoff of 500 Ry.^15^ The contracted Gaussian basis set, which includes rather diffuse primitives, is optimized by minimizing a linear combination of the total energy and the condition number of the overlap matrix. Hence, significantly fewer basis functions are required than in the usual split valence scheme while, at the same time, achieving an accuracy for the binding energies of hydrogen bonding complexes similar to the ones obtained with augmented basis sets without being plagued by the typical near linear dependencies. More specifically, no counterpoise correction had been performed, but in previous works, it had been explicitly shown that the basis set superposition error of the particular basis set for water–water and water–ammonia interactions are as low as 0.23 and 0.2 kcal/mol, respectively.^15^ Separable norm-conserving pseudopotentials were used to mimic the interactions between the valence electrons and the ionic cores.^16,17^ The B97-D exchange and correlation functional, which is based on Becke’s power-series ansatz, plus a damped atom-pairwise dispersion correction to account for long-range van der Waals interactions was employed.^18^ The K-PHI structure was modeled using a supercell with *a* = *b* = 12.5 Å, *c* = 12.8 Å and α = β = 90.0° and γ = 120.0°, which consists of 4 AA-stacked PHI-layers.^19^ Optimized structures were obtained by globally minimizing the potential energy by varying the atomic positions via dynamical simulated annealing^20,21^ based on the second-generation Car–Parrinello method of Kühne et al.^22,23^ Even though the latter is typically employed to explicitly consider finite temperature effects by means of ab initio molecular dynamics simulations, it is used here to solely locate the nuclear ground state. Yet, since the computed adsorption energies are rather large, we do not expect them to substantially change by the inclusion of entropic effects. To compute the net atomic charges, the Mulliken population^24^ and the density-derived electrostatic and chemical method DDEC6^25^ are used.

The total adsorption energies are calculated as
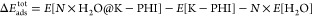
and the incremental adsorption energies as
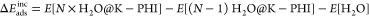
where *E*(*N* × H_2_O@K-PHI) is the potential energy of the system when *N* H_2_O molecules are adsorbed in K-PHI, whereas *E*(K-PHI) and *E*(H_2_O) are the potential energies of K-PHI and an individual H_2_O molecule, respectively. A negative value for the adsorption energy indicates that the H_2_O adsorption is thermodynamically favorable. The adsorption energy per molecule Δ*E*_ads_^mol^ is obtained by dividing the total adsorption energy by *N*. The results of Δ*E*_ads_^tot^, Δ*E*_ads_^inc^, and Δ*E*_ads_^mol^ for the H_2_O adsorption in K–PHI are shown in Table 1.

**Table 1:** Data of Multiple H_2_O

*N*(H_2_O)	Δ*E*_ads_^tot^ [kJ/mol]	Δ*E*_ads_^inc^ [kJ/mol]	Δ*E*_ads_^mol^ [kJ/mol]	wt_H2O_ [%]
1	–94.5	–94.5	–94.5	0.9
2	–207.7	–113.2	–103.8	1.8
3	–316.7	–109.0	–105.6	2.6
4	–427.3	–110.6	–106.8	3.5
5	–502.1	–74.8	–100.4	4.3
6	–584.4	–82.3	–97.4	5.1
7	–678.7	–94.3	–97.0	5.9
8	–730.0	–51.3	–91.2	6.7
9	–855.2	–125.2	–95.0	7.5
10	–971.3	–116.1	–97.1	8.3
11	–1073.8	–102.5	–97.6	9.0
12	–1174.6	–100.8	–97.9	9.8
13	–1290.1	–115.5	–99.2	10.5
14	–1327.4	–37.3	–94.8	11.2
15	–1369.2	–41.8	–91.3	11.9
16	–1414.7	–45.5	–88.4	12.6
17	–1435.5	–20.8	–84.4	13.3
**18**	**–1501.4**	**–65.9**	**–83.4**	**14.0**
19	–1478.5	23.0	–77.8	14.6
20	–1371.0	107.5	–68.5	15.3
21	–1379.0	–8.2	–65.7	15.9
22	–1245.7	133.5	–56.6	16.6

Furthermore, the electron densities ρ as well as the electron density difference upon adsorption
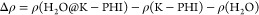
are calculated, where ρ(H_2_O@K-PHI) is the total electron density of H_2_O@K-PHI, while ρ(K-PHI) and ρ(H_2_O) are the total electron densities of K-PHI and the individual H_2_O molecule.

To investigate the nature of the interactions in H_2_O@K-PHI, the energy decomposition analysis based on the absolutely localized molecular orbital (ALMO-EDA)^26−29^ is applied. In ALMO–EDA, the total interaction energy
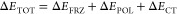
is decomposed into chemically meaningful components, such as the frozen interaction term Δ*E*_FRZ_, which is defined as the energy required to bring isolated molecules into the system without any relaxation of their molecular orbitals (apart from modifications associated with satisfaction of the Pauli exclusion principle) and an orbital relaxation contribution. The latter quantity is then further decomposed into a polarization term Δ*E*_POL_, which is defined as the energy lowering due to the relaxation of each molecule’s ALMOs in the field of all other molecules and the charge-transfer Δ*E*_CT_ contribution that is calculated as the difference in the energy of the relaxed ALMO state and the state of fully delocalized optimized orbitals. A distinctive feature of the ALMO–EDA is that the charge-transfer contribution can be separated into terms associated with forward- and back-donation for each pair of molecules, as well as a many-body higher-order (induction) contribution Δ*E*_HO_, which is very small for typical intermolecular interactions. Both the amount of electron density transferred between a pair of molecules Δ*Q*_CT_ and the corresponding energy lowering Δ*E*_CT_ can be computed via
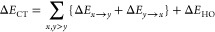
and
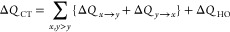


### Single Water Adsorption: Structure, Adsorption Energy, Electron Densities, and Net Charges

The calculations reveal that the adsorption of water in K-PHI is energetically favorable and a single water molecule entails an adsorption energy of Δ*E*_ads_ = −94.5 kJ/mol. This value is about twice the heat of evaporation; i.e., water is bound twice as strong to K-PHI than in its own bulk-water structure and even higher that the heat of dilution of sulfuric acid with water.^30^ This also strongly supports the experimental observations. The reason why water is bound more strongly at the surface of K-PHI than in bulk water is due to the presence of negatively charged surfaces in the crystal. The surface layers with the water molecules through electrostatic forces lead to the formation of an ordered water layer at the interface. This ordered layer is commonly referred to as the “hydration layer” or “solvation shell”, a physically different zone with different water. The strength of the interaction between water molecules and the salt surface is determined by the nature and magnitude of the electrostatic forces. Our negatively charged pore surface in the structure attracts the positively polarized hydrogen atoms in water molecules, resulting in the unusually strong hydrogen bond between the water molecule and the surface and the related higher binding energy for water bound to the pore surface as compared to bulk water.

**Figure 1:**
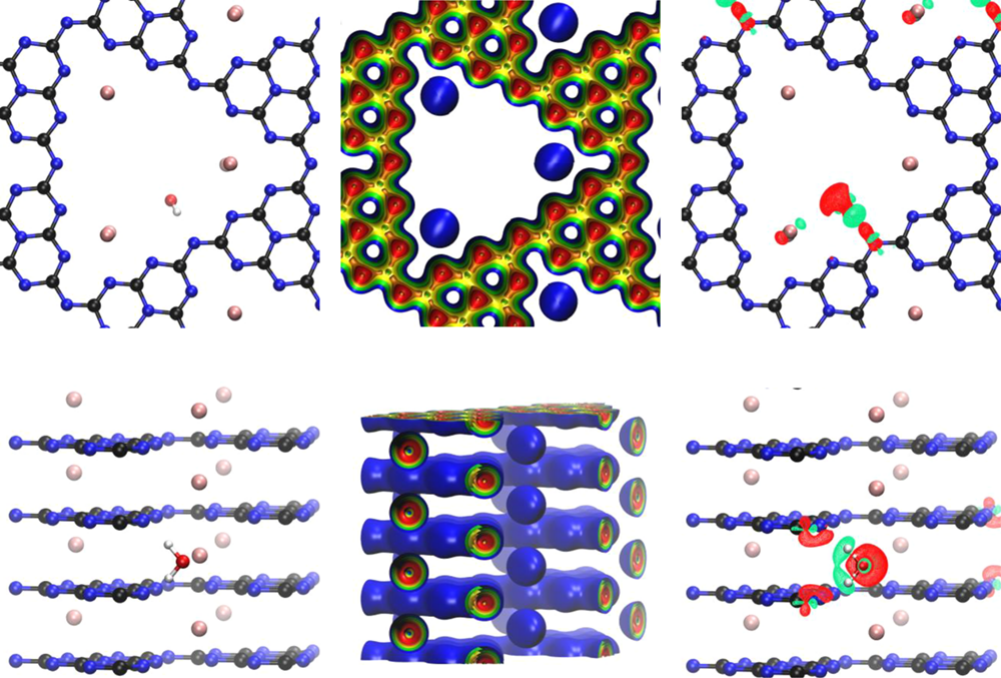
Adsorption state of a single water molecule in K-PHI (left), electron density isosurfaces of K-PHI (middle, isovalues = 0.05/0.1/0.2/0.3/0.4 from blue to red), and the electron density differences upon water adsorption on K-PHI (isovalues = ± 0.002 red(+)/green). Top: top view, bottom: side view. Atom colors: K = pink; C = black; N = blue, O = red, H = white.

The adsorption site of the water molecule in K-PHI lies in the pores between the PHI layers, as depicted in Figure 1. In that case, water forms one hydrogen bond each with the surface nitrogen atoms of both surrounding PHI layers. The distances between the hydrogen atoms of the water and the involved nitrogen atoms are 2.20 and 2.07 Å, respectively.

To investigate the electronic environment of the adsorbed water, net atomic charges were calculated using the Mulliken population analysis and the density-derived electrostatical and chemical method DDEC6. The averaged values for each atomic kind as well as the summed values for H_2_O and the PHI layers are summarized in Table 2. The negative charge of the PHI system (formally −3, Mulliken −1.8, DDEC6 −2.45) is distributed throughout the PHI system but mostly located at the nitrogen atoms, which are more electronegative when compared to the carbon atoms. In the pristine K-PHI, this is compensated by partial positive charges at the potassium ions. This behavior can be nicely observed in the top view of the electron density distribution of pristine K-PHI in Figure 1 (middle), where the highest electron densities (red) are around all the nitrogen atoms. In the adsorbed state, water carries a slightly negative charge due to charge transfer from the PHI system to the water. This is also illustrated in the electron density difference representations in Figure 1 (right), where the charge is decreased around the surface nitrogen atoms. A more precise description of the interaction is discussed in the following energy decomposition analysis.

**Table 2:** Averaged Net Charges Calculated by Mulliken as well as the DDEC6 Method of a Single Water Molecule Adsorbed on K-PHI

	Mulliken	DDEC6
K	+0.61	+0.83
C	+0.10	+0.55
N	–0.17	–0.54
O	–0.34	–0.84
H	+0.13	+0.36
PHI layer	–1.81	–2.45
H_2_O	–0.07	–0.12

The fact that water is not directly bound to K^+^ ions is a particularity of the hydration structure of the alkali metals. Contrary to the Na^+^ hydration shell, which is formed by three water molecules, K^+^ can only perturb the water structure in its immediate neighborhood so that it is unable to attract the water molecules. If indeed K^+^ would disturb the water structure, we would see exactly that in the simulations.

### Energy Decomposition Analysis by Absolutely Localized Orbitals (ALMO-EDA)

To study the interaction between water and the PHI system more closely, the ALMO-EDA method is applied to the single water adsorption, which allows one to decompose the interaction between the water molecule and the K-PHI system into physically illustrative components, i.e., frozen energy Δ*E*_FRZ_, polarization energy Δ*E*_POL_, and charge transfer energy Δ*E*_CT._
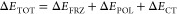


The decomposition of the interaction energy leads to the results presented in Table 3:

**Table 3:** ALMO-EDA-Based Energy Decomposition of the Interaction between Water and K-PHI

	Δ*E*_FRZ_	Δ*E*_POL_	Δ*E*_CT_
H_2_O @K-PHI	–57.4 kJ/mol	–18.8 kJ/mol	–42.8 kJ/mol

The difference between the calculated adsorption energy (−94.5 kJ/mol) and the sum of the ALMO-EDA interactions (−119.0 kJ/mol) originates by slight distortion of the water molecule and repositioning of potassium cations in the adsorption state compared to their individual optimized structures. This energy penalty is not included in the ALMO-EDA calculation and sometimes referred to as geometric distortion term.^31^

The polarization interaction is indeed comparably low (as expected from the bulk water index of refraction) and is in the correct as well as expected range of the polarization interaction of a rather unpolarizable molecule to a high refractive index surface. The frozen energy includes the H-bridge interactions and gives a slightly higher value than evaporation enthalpy; that is, the only two H-bridges are significantly stronger than the up to four ones in regular bulk water.

**Figure 2:**
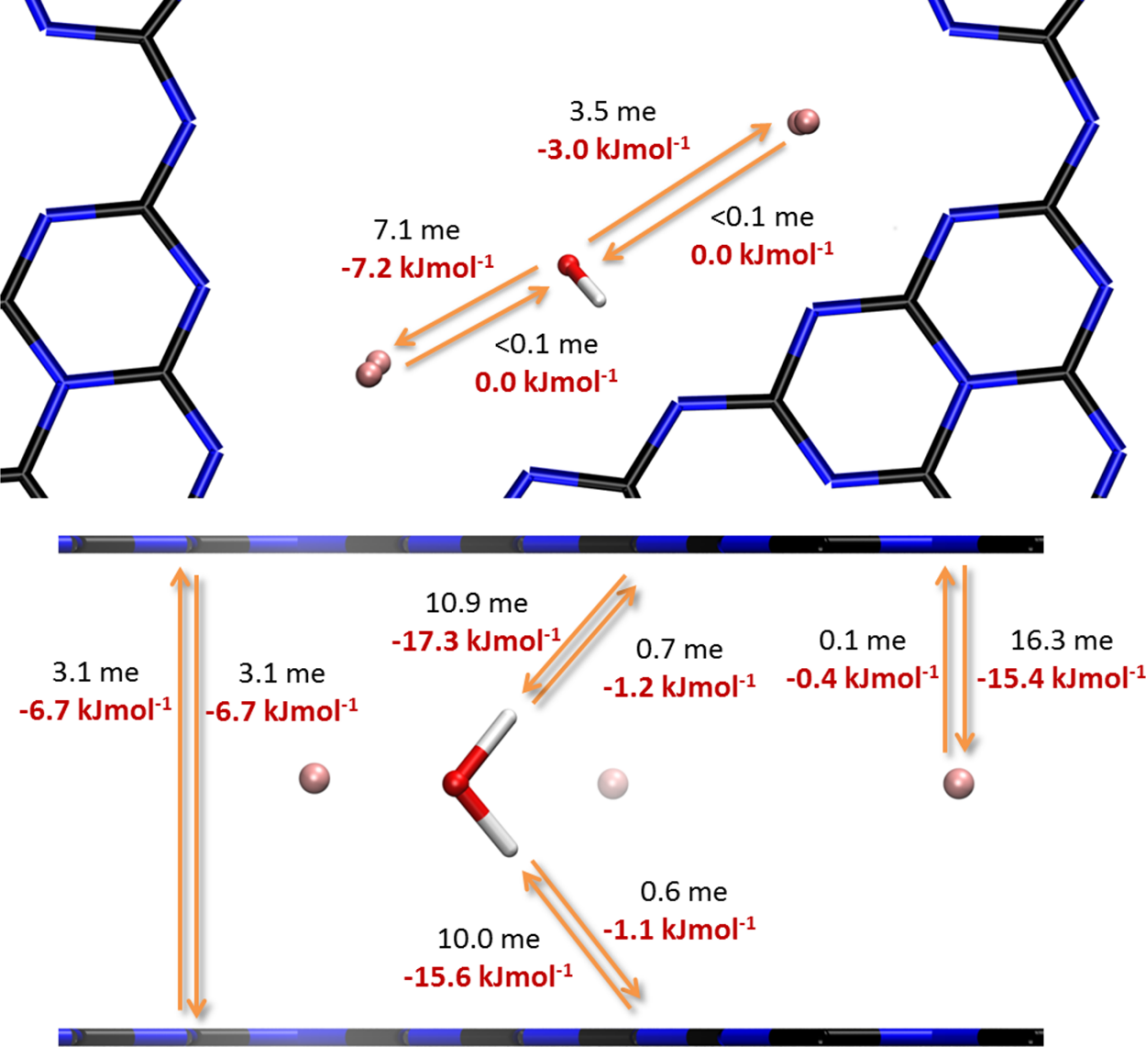
Horizontal (top) and vertical (middle) charge transfers (black) occurring between water, the positive K^+^, and the negative PHI layers as well as the resulting stabilization energy (red) upon single water adsorption in K-PHI as computed by the ALMO-EDA. Atomic colors: K = pink; C = black; N = blue, O = red, H = white.

A high part (−42.8 kJ/mol) of the extra interaction between the water molecule and the adsorbent is however due to charge transfer, which can be further decomposed in order to obtain the acceptor and donor contributions. All charge transfers and resulting stabilization energies are shown in Figure 2.

The largest contributions of the CT can be identified as charge transfer from the two neighboring PHI layers to H_2_O (in total: −32.9 kJ/mol, 20.9 me), seconded by the transfer from H_2_O to the nearest K^+^ ions (−10.2 kJ/mol, 10.6 me). The values of the average net charges before water adsorption in the material are essentially identical and thereby not discussed separately, which is why they are not shown. This is further circumstantiated by the fact that the average net charge of water itself is rather small.

### Multiple Water Adsorption: Adsorption Energies and Structures

**Figure 3:**
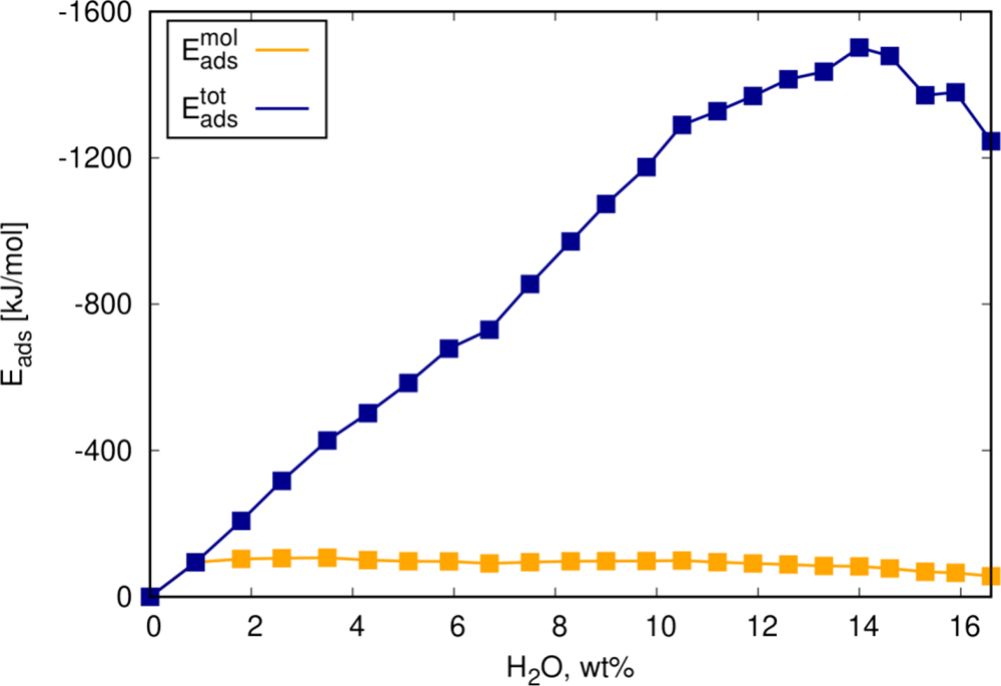
Total adsorption energy Δ*E*_ads_^tot^ of water in K-PHI as a function of water loading to determine the maximum H_2_O uptake as well as the adsorption energy per molecule Δ*E*_ads_^mol^.

**Figure 4:**
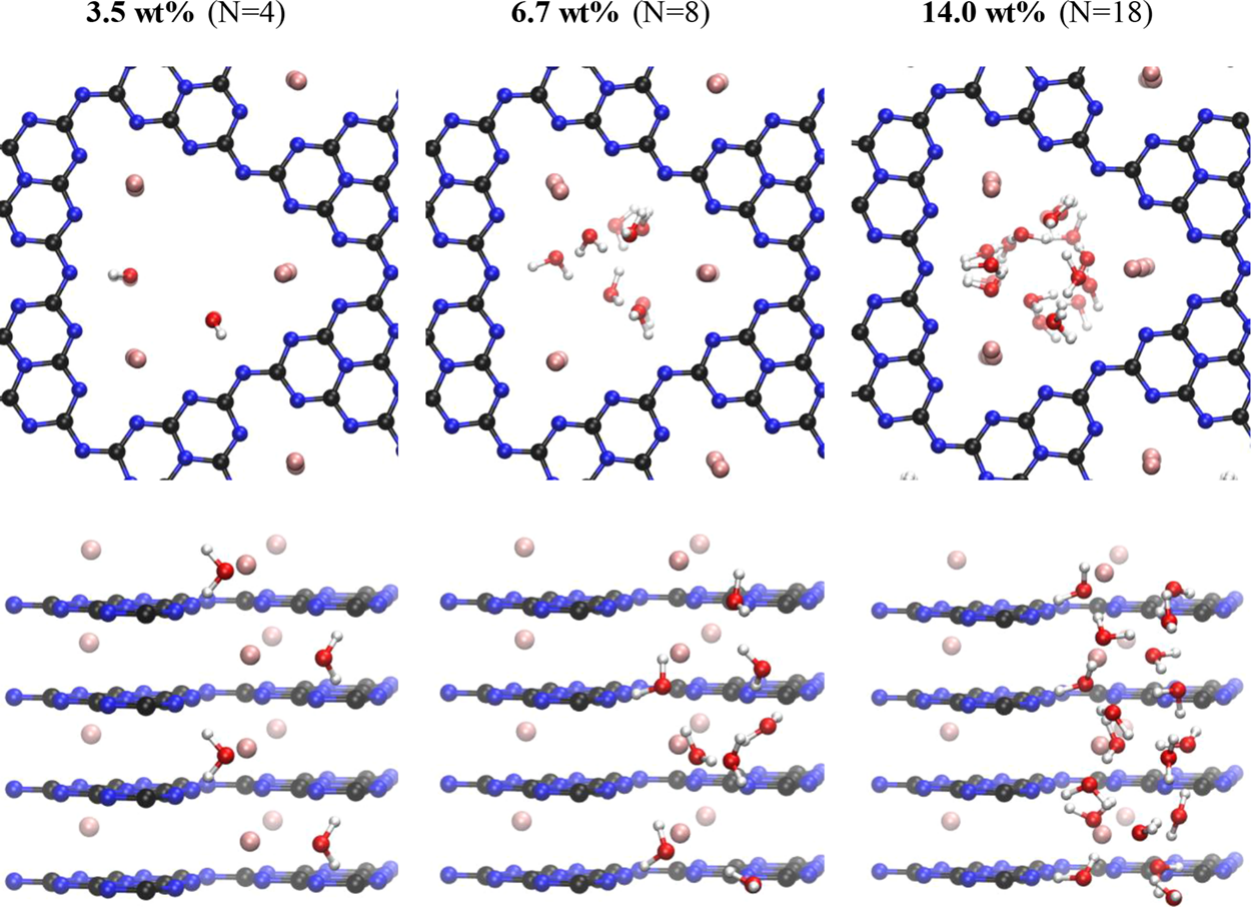
Adsorption states of water in K-PHI at different H_2_O loadings. N is the number of water molecules inside the supercell. Top: top view, bottom: side view. Atomic colors: K = pink; C = black; N = blue, O = red, H = white.

The interaction with single water molecules is just the starting point, in a physico-chemical view the nucleation of a liquid water cluster at the surface. Of course, K-PHI then adsorbs multiple additional water molecules with similar high incremental adsorption energy. The structure of the adsorbed water molecules then depends on the amount of water. The first generation of adsorbed water molecules on K-PHI are well organized, and the adsorption state is similar to the individual water adsorption until one water molecule per pore and per layer is reached (Figure 3; 3.5 wt % corresponding to N = 4 H_2_O molecules in the simulation). Throughout additional adsorption, water molecules also tend to form hydrogen bonds with each other, resulting in a nanocluster structure, but still mostly at similar adsorption sites and instrumentalizing the higher hydrogen bond strength toward the PHI layer, as shown in Figure 4 at 6.7 wt %. The incremental adsorption analysis reveals that K-PHI can adsorb water until a final uptake of 14.0 wt % (*N* = 18 per pore) and a maximum Δ*E*_ads_^tot^= – 1501 kJ/mol are reached (Figure 3). Hence, the final adsorption energy per molecule upon complete filling even in the cluster is Δ*E*_ads_^mol^= −83.4 kJ/mol per H_2_O. As the relative importance of the stronger PHI-water interactions decreases, the adsorption energy per molecule decreases.

**Figure 5:**
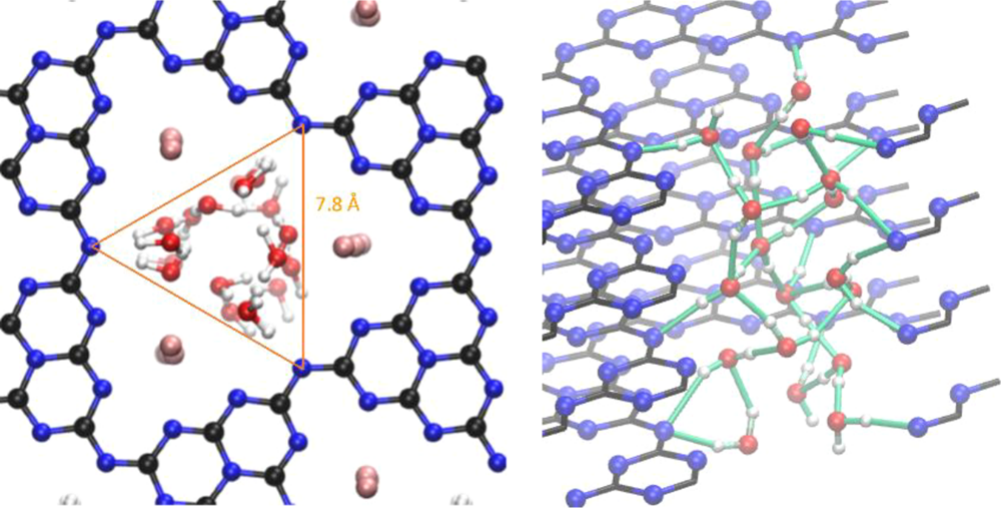
Illustration of the confined space, where water is located inside these pores as a rough estimation for the occupied volume of water (left), and view of the hydrogen bond network of water in K-PHI, where water forms hydrogen bonds with other water molecules as well as the surface nitrogen atoms of the PHI walls. Atomic colors: K = pink; C = black; N = blue, O = red, H = white, hydrogen bonds = green.

The interesting point concerning water structure in nanosized pores is that charge donation and acceptance propagates through the water cluster network and modifies the whole structure and binding strengths. The structure is liquid, and the symmetry follows essentially a trigonal pore symmetry and the three strong anchoring sites (Figure 5). By simplifying the complex structure, we could describe the geometry as a water nanotube, with the typical water cage pores being aligned toward the center of the K-PHI pore. The secondary hydrogen bridges between the water molecules are largely aligned perpendicular to the layered PHI structure, very similar to the natural master pattern of aquaporines,^32^ but with a triple water construction. That is, the model is potentially even able to reason on the excellent transport properties of water and protons through such carbon nitride layers.^33,34^

Determining the density of the water is not straightforward as the occupied volume and definition of accessible space or pore volume have a crucial influence on the formal density in these narrow pores. If we consider the occupied volume upon complete adsorption as the space confined by the triangle between the bridging nitrogen atoms within the PHI layer, which is an arbitrary definition and shown in Figure 5 (left), we obtain a density of around 1600 kg/m^3^. This might be too high as there are some water molecules still on the edge of the triangle; however, it clarifies that water only occupies a very small space, which is around 30% of the total volume because of the present potassium cations. Increasing the volume by an educated guess from known oxygen radii leads to lower densities down to 1100 kg/m^3^, which is still significantly higher than the density of bulk water.

An alternative approach is to compare the intermolecular distances of hydrogen-bonded water molecules in the pores to the ones of bulk water. We find 14 HO–H···N-R hydrogen bonds of H_2_O with the wall surface with an average distance of 1.90 Å as well as 22 HO–H···OH_2_ hydrogen bonds between water molecules with an average distance of 1.73 Å. All 36 hydrogens of the 18 water molecules could hence be assigned to a hydrogen bond. The hydrogen bonding network is shown in green in Figure 5 (right). The water–water coordination number is only 1.2 because water highly interacts with the pore walls and the present potassium cations. The intermolecular O–O distances between the 22 hydrogen-bonded water pairs are on average 2.66 Å. This distance is massively lower than the most frequent O–O distance in the first shell of bulk water, which is around 2.79 Å.^35^

A by 5% shortened distance between the oxygen constituents of course comes with a 10% increased density of the bent water monolayer film in the pores and a massively increased cohesion energy when compared to bulk water; i.e., the data are even semi-quantitatively self-consistent. This is pleasing as the model suggests that we can relate the experimentally easily accessible O–O distance data (from the amorphous water scattering) directly to thermodynamic properties of water.

## Conclusions

Modeling of water molecules into the nanopores of K-PHI, a 2D covalent material with experimentally reported strong water interactions, gave some exciting details on the structure and thermodynamics of this process. First, water molecules are bound with −94.5 kJ/mol, which is twice the cohesion energy of water and one of the highest adsorption enthalpies reported so far. The strong binding, however, proceeds with only slightly lower enthalpies until the pore is filled, while the binding strength is passed through the conjugated water h-bridge network, as expressed in strongly shortened O–O distances which are on average about 5% shorter than in bulk water. This also corresponds to a higher water density, which for a 2d structure was estimated to be above 1.1 g/ cm^3^. The H-bridges are strongly aligned in the direction perpendicular to the covalent planes, which could describe the observed high ion fluxes and conductivity of such membranes.

Decomposition of the adsorption energy into components reveals an expectedly low polarization interaction but an unexpectedly high charge transfer contribution, where the partly naked K^+^ ions play a key role. This not only offers a new structural lead motif for the design of stronger but reversible adsorption materials involving metal ions on their surface but also puts metal cations as known cofactors in biochemistry into a more central role of even more remote molecular interactions.
